# Carbohydrate status in patients with phenylketonuria

**DOI:** 10.1186/s13023-018-0847-x

**Published:** 2018-06-27

**Authors:** María L. Couce, Paula Sánchez-Pintos, Isidro Vitoria, María-José De Castro, Luís Aldámiz-Echevarría, Patricia Correcher, Ana Fernández-Marmiesse, Iria Roca, Alvaro Hermida, Miguel Martínez-Olmos, Rosaura Leis

**Affiliations:** 1 Unit of Diagnosis and Treatment of Congenital Metabolic Diseases, S. Neonatology, Department of Pediatrics, Hospital Clínico Universitario de Santiago de Compostela, CIBERER, Health Research Institute of Santiago de Compostela (IDIS), A Choupana, s/n, 15706 Santiago de Compostela, A Coruña, Spain; 2 Unit of Metabolopathies, Hospital Universitario la Fe, Bulevar sur s/n, 46021 Valencia, Spain; 3 Unit of Metabolism, Department of Pediatrics, Hospital de Cruces. Group of Metabolism, Biocruces Health Research Institute, CIBERER, Plaza de Cruces s/n, 48903 Barakaldo, Vizcaya Spain; 4 Unit of Gastroenterology and Nutrition, Department of Pediatrics, Hospital Clinico Universitario de Santiago, IDIS, Travesía da Choupana s/n,15706 Santiago de Compostela, A Coruña, Spain

**Keywords:** HOMA index, Insulin, Nutrition, Phenylalanine, Tetrahydrobiopterin

## Abstract

**Background:**

In patients with phenylketonuria (PKU), a low-phenylalanine (Phe) diet supplemented with low-protein foods and a Phe-free amino acid mixture favors a dietary intake rich in carbohydrates, but little is known about how these molecules are metabolized in this setting. The objective of the present study was to analyze carbohydrate metabolism in patients with hyperphenylalaninemia.

**Methods:**

We conducted a multicenter cross-sectional study to investigate biochemical markers of basal and postprandial carbohydrate metabolism in PKU patients according to age, Phe tolerance, waist circumference and body mass index (BMI), diet, tetrahydrobiopterin (BH4) supplementation, and adherence to treatment. Basal biomarkers and anthropometric parameters were also evaluated in patients with mild hyperphenylalaninemia (MHPA) and in healthy controls.

**Results:**

A total of 83 patients aged 4–52 years were studied; 68.7% had PKU and 31.3% had MHPA. 68 healthy controls of similar sex and age were also evaluated Metabolic control was adequate in 71.9% of PKU patients. Fasting glucose levels (mean 80.77 ± 8.06 mg/dL) were high in just one patient, but fasting insulin levels, with a mean of 12.74 ± 8.4 mIU/L, were altered in 15 PKU patients (26.3%) and markedly higher than in patients with MPHA (*p* = 0.035). Fasting insulin levels and Homeostasis Model Assessment Insulin Resistance (HOMA-IR) were significantly higher than in healthy controls and correlated with body mass index, waist circumference, age, and also showed statistically significant differences according to diagnosis and Phe tolerance (*p* < 0.05). Patients under BH4 therapy had lower insulin levels and HOMA-IR. A higher mean carbohydrate intake from AA mixtures was observed in classic PKU patients. The caloric intake in the form of carbohydrates was also higher in PKU than MHPA patients (*p* = 0.038) and it was correlated with basal insulin (rho = 0.468, *p* = 0.006), HOMA-IR (rho = 0.423, *p* = 0.02), BMI (rho 0.533, *p* = 0.002), and waist circumference (rho 0.584, *p* = 0.0007).

**Conclusions:**

This study shows that PKU patients are at risk of carbohydrate intolerance and insulin resistance, more evident in adults and overweight patients, probably related to their higher caloric intake in form carbohydrate content. A higher dependency of AA mixtures was demonstrated in PKU patients.

**Electronic supplementary material:**

The online version of this article (10.1186/s13023-018-0847-x) contains supplementary material, which is available to authorized users.

## Introduction

Phenylketonuria (PKU) is an autosomal recessive disorder of phenylalanine (Phe) metabolism caused by deficient activity of the hepatic enzyme L-phenylalanine-4-hydroxylase (PAH, EC 1.14.16.1) due to variations in the PAH gene (NM 000277.1) (OMIM 261600). The estimated overall incidence of PKU in Caucasians is 1 case in every 10,000 live births [1]. PKU is usually detected by newborn screening, enabling early diagnosis and treatment and consequently prevention of serious, irreversible neurological sequelae. Treatments aimed at maintaining optimal serum Phe concentrations include dietary treatment and a synthetic form of tetrahydrobiopterin (BH4) in patients who respond to the BH4 loading test [2–4]. Most patients, however, receive dietary treatment only [5]. The standard PKU diet is a low-Phe diet, which consists of restricted natural protein intake supplemented with special low-protein foods and a Phe-free amino acid mixture (AA mixture). Although dietary treatment has been used in PKU for over 50 years, its metabolic impact remains to be optimized [6–8]. There has been increasing interest in improving the nutritional profile of AA mixtures to ensure nutritional adequacy and optimal growth [7, 9, 10]. Moreover, the nutritional profile of these mixtures varies considerably from one product to the next [11] and this is something that needs to be addressed if long-term outcomes are to be improved. Altered micronutrient status [9], vitamin deficiencies [12] and an atherogenic lipid profile in individuals with excess weight [13] have all been described in PKU.

Despite the risks associated with high carbohydrate intake, little research has been done on carbohydrate metabolism in patients with PKU. According to one recent study, children with PKU consumed more carbohydrates (% of total energy) than healthy controls with a higher dietary glycemic index [14]. The authors claimed that the restriction of natural proteins favors, and might even physiologically stimulate, the consumption of foods rich in carbohydrates (in particular, simple carbohydrates), leading to a higher glycemic index diet. PKU patients are thus vulnerable to metabolic abnormalities and excess weight [15].

Patients who adhere to dietary recommendations appear to have similar cardiovascular risks to the healthy population [16]. However, risk factors such as an atherogenic lipid profile, elevated blood pressure, and high-sensitivity C-reactive protein concentrations have been reported in overweight or obese patients with PKU [13]. Considering the shortage (and small size) of studies analyzing carbohydrate metabolism in patients with PKU [14, 15, 17, 18], we undertook a cross-sectional study of children and adults with PKU from three Spanish hospitals to analyze biochemical markers of basal and postprandial carbohydrate metabolism according to Phe tolerance, age, waist circumference (WC), body mass index (BMI), diet, BH4 supplementation, and adherence to treatment. We also analyzed the potential role of ghrelin and HOMA-IR as cardiometabolic risk factors in this population.

## Patients and methods

### Study design

This was a cross-sectional observational study conducted from February 2016 to April 2017Written informed consent was obtained from all patients or from parents or legal guardians in the case of children < 16 years of age. The population comprised patients diagnosed with PKU at three Spanish hospitals. All the patients were monitored using the same protocol at their respective hospital. Patients were diagnosed through newborn screening (introduced for PKU in Spain in the 1970s–1980s) or clinical suspicion. Exclusion criteria were poor medical monitoring, changes to AA mixture in the month prior to inclusion in the study, and pregnancy. A control group of 68 healthy cases, paired by age and sex, were also included.

### Parameters

The following parameters were evaluated: age (adults vs. patients ≤18 years); sex; phenotype (mild hyperphenylalaninemia [MHPA] [serum Phe level 120–360 μmol/L at diagnosis], mild–moderate PKU [MPKU] [360–1200 μmol/L], or classic PKU [CPKU] [> 1200 μmol/L] according to US guidelines [19]); disease detection (early vs. late diagnosis); annual median blood Phe levels (target levels of 120–360 μmol/L for children < 12 years and 120–600 μmol/L for older patients as established in Spanish recommendations published in 2014 [20], which are also congruent with the most recent European guidelines for PKU [21]); carbohydrate content in AA mixture; HOMA-IR; anthropometric data (weight, height, WC, and BMI), Phe tolerance (low < 500 mg/day vs. high > 500 mg/day); 6R-BH_4_ therapy (yes vs. no). We also assessed the following blood biochemical markers: glucose, insulin, plasma amino acids (Phe, tyrosine, alanine, valine, leucine, isoleucine, and threonine), C-peptide, urea, ghrelin, insulin-like growth factor 1 (IGF1), IFG-binding protein 3 (IGFBP3), lactate, pyruvate, and fructosamine (this parameter was only was evaluated in one center). Metabolic control, or dietary adherence, was evaluated using median blood Phe levels for the last year and the pre-established “safe” thresholds for each age group (see above). Basal blood samples were collected at the same time each day (at 9:00 after overnight fasting) in all patients and 120 min after ingestion of the adjusted AA mixture in the case of patients with MPKU and CPKU. Blood was not collected during infections or treatment with medication other than 6R–BH_4._ Questions about these aspects were formulated prior to taking samples. The reference ranges for the biochemical markers were as follows: glucose (74–105 mg/dL), insulin (1.5–18.5 mIU/L), peptide C (0.81–2.85 ng/mL), urea (13–50 mg/dL), ghrelin (520–700 pg/mL), IGF1 (66–249 mg/mL), IGFBP3 (3–7 μg/mL), lactate (0.63–2.44 mmol/L), pyruvate (0.033–0.077 mmol/L), and fructosamine (151–300 μmol/L).

### Methods

The three hospitals followed the same protocol. Dietary treatment was based on Spanish PKU guideline recommendations [22] and consisted of restricted natural protein intake supplemented with an AA mixture. Mean protein intake was 1.3–1.5 times higher than recommended dietary allowances [23]. The AA mixture was taken three or four times a day, with one-third of the total dose being taken in the morning. The diet was assessed weekly using 3-day food surveys completed online (www.odimet.es). Patients with MHPA at diagnosis did not require treatment and followed a normal diet.

Standing height was measured using a wall-mounted stadiometer and patients were weighed (barefoot and after overnight fasting) to the nearest 100 g on a digital scales. Nutritional status according to BMI was calculated as weight (kg) /height^2^ (m^2^). Patients > 18 years of age were classified according to the WHO criteria into four categories: underweight (BMI < 18.5), normal weight (BMI 18.5–24.99), overweight (BMI 25–29.99), and obese (BMI ≥30). Younger patients were classified using the WHO Child Growth Standards as underweight (BMI <15th percentile), normal weight (BMI 15-85th percentile), overweight (BMI 85-95th percentile), or obese (BMI >95th percentile) [24, 25].

WC was measured in centimeters midway between the lower rib margin and the iliac crest. In the case of children, WC measurements were stratified by sex and age following the method described in the Galinut Study [26]. For adults, specific WC values from the International Diabetes Federation were used [27].

- HOMA-IR was calculated using the Matthews formula [28]: insulin (μU/mL)* [glucose (mmol/L)] / 22.5], with a score ≥ 2.5 indicating insulin resistance [29]. Insulin resistance was also assessed using the quantitative insulin sensitivity check index (QUICKI): 1/log10 basal insulin (uIU/mL) + log10 basal glucose (mg/dL) [30].
- Glucose, urea, and fructosamine levels were measured by colorimetric enzymatic analysis (ADVIA 2400, Siemens) and lactate and pyruvate were measured using a quantitative Trinder method assay (BEN S.r.l). Serum insulin and C-peptide levels were measured using the ADVIA Centaur XP immunoassay system (Siemens), while IG 1 and IGFBP3 were measured using the chemiluminescence IMMULITE 2000 immunoassay system (Siemens). Total serum ghrelin was determined by radioimmunoassay (Ghrelin Total RIA Kit, Linco Research). Quantitative analysis of plasma amino acids was performed using ion-exchange chromatography (Biochrom 30 Amino Acid Analyzer) after deproteinization of samples with 5-sulfosalicylic acid.

### Statistical analysis

To determine significant associations and/or differences between the study variables, we first used the Kolmogorov–Smirnov and Shapiro–Wilk tests to check for normal distribution. In cases where one of the variables was quantitative and the other qualitative, we applied the Student t-test or ANOVA if the quantitative variable was normally distributed, and the Wilcoxon signed-rank test or the Kruskal-Wallis test otherwise. The Fisher exact test was used when both variables were qualitative. To assess correlations between quantitative variables, we used Pearson’s correlation for normally distributed data and Spearman’s rho otherwise. The resulting *p* values were adjusted using the Benjamini-Hochberg procedure and only values lower than 0.05 were considered significant. To evaluate interdependencies between variables a stepwise regression method based on AIC (Aikake Information Criterion) was used; the adjusted model was a multiple or linear regression when the response variable was quantitative, and a logistic regression model for two-level qualitative variables. Statistical analysis was performed using R Core Team (2017), version 3.4.1. [31]

## Results

Eighty-three patients (40.96% male; age range 4–52 years; 48.2% adults) were included in the study. Of these, 57 (68.7%) had PKU and 26 (31.3%) had MHPA. PKU was classified as CPKU group in 37 patients (64.9%) and as MPKU in 20 (35.1%). Seventy patients (84.3%) were diagnosed through newborn screening. Ten patients (17.5%) with PKU received 10–20 mg/kg/day of 6R-BH4 therapy and all of them also received dietary treatment but relaxed (Table 1). In the control group there were no differences of sex (45.6% males, *p* = 0.85) or age (mean: 19.34 ± 12.45; *p* = 0.27).

**Table 1:** Characteristics of patients according to type of diagnosis

Variables	Type of diagnosis			*p* ^*1*^	*p* ^*2*^
	CPKU	MPKU	MHPA		
Patients N	37	20	26		
Age (y)	24.92 ± 11.64	16.7 ± 7.26	13.23 ± 10.24	0.002	0.0006
Sex (F/M)	19F / 18 M	13F / 7 M	17F / 9 M	0.48	0.47
TD (E/L)	27E / 10 L	18E / 2 L	25E / 1 L	0.38	0.26
Treatment (D/P + D)	36D / 1P + D	11D / 9P + D	–	–	–
BMI (kg/m^2^)	25.57 ± 5.96 15Nor/22↑	22.12 ± 5.78 14Nor/6↑	18.46 ± 3.79 22Nor/4↑	0.0003	0.0004
WC (cm)	87.08 ± 17.42 13Nor/24↑	73.96 ± 13.92 16Nor/4↑	64.85 ± 11.59 22Nor/4↑	0.002	0.001
Phe median inter- quartile range (μM).	484(378–840)	241.9(205–482)	295.5(246–310)	0.007	3.2e^−05^
Phe tol (mg/day)	379 ± 148.57	868.9 ± 682.53	Free diet-	–	–
Glucose (mg/dL)	81.24 ± 9.76	78.05 ± 4.12	82.19 ± 7.4	0.29	0.29
Insuline (mUI/L)	16.22 ± 10	11.12 ± 5.72	9.03 ± 5.34	0.035	0.02
Peptide C (ng/mL)	2.3 ± 1.03	1.54 ± 0.78	1.31 ± 0.72	0.028	0.002
Urea (mg/dL)	21.86 ± 6.14	23.3 ± 6.36	26.15 ± 6.58	0.18	0.31
Fructosamine(μM)	234.25 ± 44.36	222.8 ± 25.2	232.65 ± 32.74	0.7	0.28
Lactate (mM)	1.28 ± 0.61	1.01 ± 0.49	1.29 ± 0.65	0.45	0.31
IGF-1 (mg/mL)	204.77 ± 77.7	227.89 ± 112.34	193.35 ± 125.69	0.11	0.22
IGF-BP3 (μg/mL)	4.68 ± 1.2	4.65 ± 1.39	4.39 ± 0.97	0.34	0.62
Ghrelin (pg/mL)	884.94 ± 454.91	687.5 ± 319.34	977 ± 517.78	0.16	0.25
HOMA-IR	3.35 ± 2.26	2.17 ± 1.17	1.88 ± 1.19	0.034	0.037
QUICK-Index	0.33 ± 0.03	0.35 ± 0.04	0.36 ± 0.04	0.019	0.026
Total intake of carbohydrates per day (g)	282.59 ± 68.9	248.23 ± 91.94	206.4 ± 47.64	0.17	0.31
% total Kcal as carbohydrates	57.04 ± 8.55	53.47 ± 9.8	45.4 ± 7.4	0.038	0.046

N: sample size; CPKU: classic PKU; MPKU: mild-moderate PKU; MHPA: mild hyperphenylalaninemia; y: year; TD: time of diagnosis; L: late diagnosis (by clinical symptoms or familiar history, after first month of life), E: early diagnosis (by newborn screening); Treatment: D: dietary treatment; P: pharmacological treatment; BMI: body mass index; WC: waist circumference; nor. Normal; *p*^*1*^: *p* value of the comparison between PKU (CPKU+MPKU) and MHPA; *p*^*2*^: *p* value of the comparison between CPKU, MPKU and MHPA

Adequate metabolic control was observed in 41 (71.9%) of the 57 PKU patients over the observation period; in 18 (54.5%) of the 33 adult PKU patients, and in 100% of those who received 6R-BH4 treatment. In the overall group, BMI was above the upper limit in 32 patients (38.6%), 75% of whom were overweight and 25% of whom were obese. Thirty-two patients (38.6%) had WC values above the upper limit (>95th percentile in 10 cases). Compared with the MPHA group, a significantly higher proportion of patients in the PKU group had BMI and WC above the upper limit (87.5% vs. 12.5%, *p* = 0.0062). This was also the case for adults (68.75% vs. 21.25% for BMI, *p* = 0.010 and 71.9% vs. 28.1% for WC, *p* = 0.0011)**.** BMI above the upper limit was significantly more common in patients with good rather than poor metabolic control (68.75% vs. 31.25%, *p* = 0.044) (Additional file 1). Moreover, a higher percentage of BMI above the upper limit was observed in PKU patients with late diagnosis and treatment (90%, 9/10) when compared with those with early-diagnosis (48.1%, 13/27) (*p* = 0.023). In the control group of 68 healthy cases (age range: 4–49 years), 25% (17 cases: 10 obese and 7 overweight) had a BMI above the upper limit (Additional file 2).

**Fig. 1:**
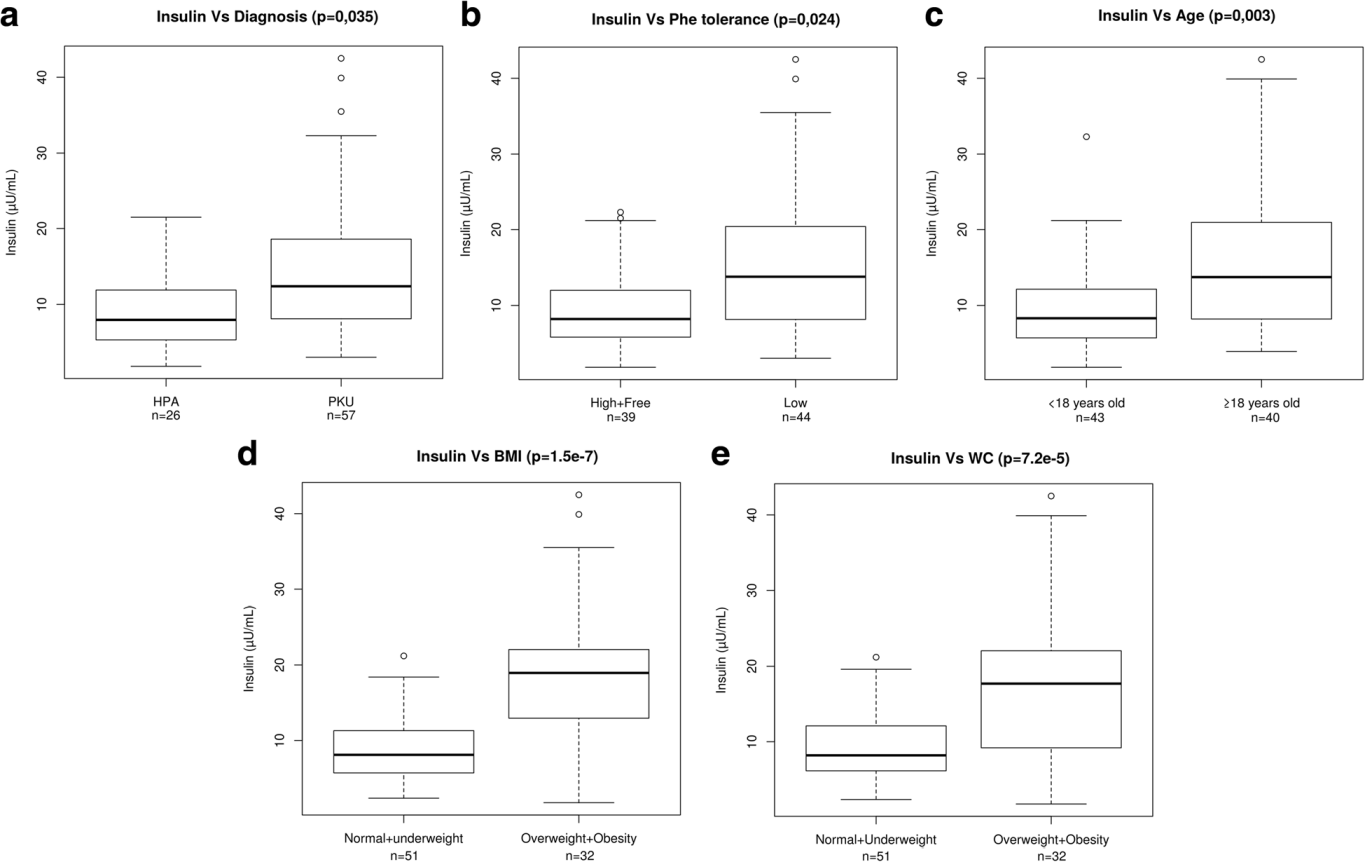
Differences in basal insulin levels (mIU/L) according to **a** type of diagnosis (PKU vs MHPA); **b** Phe tolerance (high or free vs low); **c** age (under 18 years vs over 18 years); **d** BMI (normal and underweight vs overweight and obesity); and **e** WC (normal and underweight vs overweight and obesity). PKU: phenylketonuria; MHPA: mild hyperphenylalaninemia; Phe: phenylalanine; BMI: body mass index; WC: waist circumference; n: sample size; p: *p*-value

**Fig. 2:**
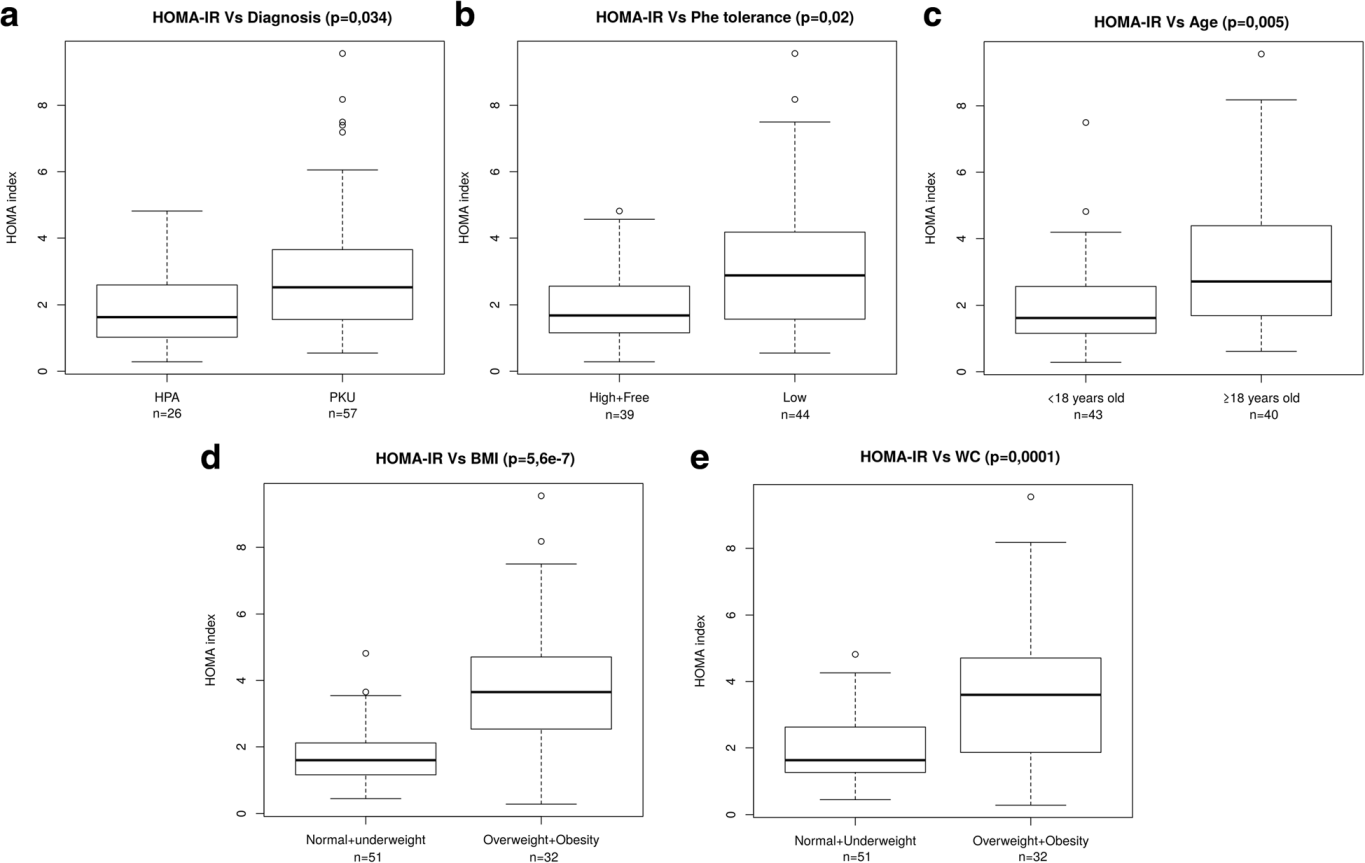
Differences in HOMA-IR scores according to **a** type of diagnosis (PKU vs MHPA); **b** Phe tolerance (high and free vs low); **c** age (under 18 years vs over 18 years); **d** BMI (normal and underweight vs overweight and obesity); and **e** WC (normal and underweight vs overweight and obesity). HOMA-IR: homeostasis model assessment insulin resistance; PKU: phenylketonuria, MHPA: mild hyperphenylalaninemia; Phe: phenylalanine; BMI: body mass index; WC: waist circumference; n: sample size; p: p-value

The mean fasting glucose level was 80.77 ± 8.06 mg/dL (range 64–111 mg/dL) and only one patient had high fasting glucose levels. Fasting insulin levels, with a mean of 12.74 ± 8.4 mIU/L (range 1.8–42.5), were above upper limit in 15 PKU patients (26.3%) and 3 MHPA patients (11.54%) and were markedly higher in the former group (*p =* 0.035) (Table 1). Fasting insulin levels correlated with BMI (rho = 0.72), WC (rho = 0.55), and age (rho = 0.463) (Table 2), and they also showed statistically significant differences according to diagnosis and Phe tolerance (Fig. 1). Mean fasting HOMA-IR for the overall group was 2.6 ± 1.8 (range 0.28–9.54; in children: 2 ± 1.38 and in adults: 3.25 ± 2.08) and, like insulin, was positively related to the same biochemical markers of carbohydrate metabolism (Table 2, Fig. 2). Conversely, patients under BH4 treatment had lower insulin levels (9.9 ± 5.4 mIU/L vs. 13.1 ± 8.6 mIU/L) and HOMA-IR (1.92 ± 1.1 vs. 2.69 ± 1.9).

**Table 2:** Correlation (Rho) of carbohydrate metabolism markers with age, Phe tolerance, Phe anual median levels, body mass index, waist circumference and caloric intake in the form of carbohydrates in hyperphenylalaninemia patients

	AGE (years)	Phe tolerance (mg)	Phe median levels (adequate to age)	BMI (Kg/m^2^)	WC (cm)	Caloric intake in the form of carbohydrates
Basal Insulin (mIU/L)	0.463 *p* = 8.5e^−5^	−0.164 n.s.	0.38 *p =* 0.003	0.728 *p* = 1.5e^−12^	0.557 *p* = 1.4e^−5^	0.468 *p* = 0.006
HOMA IR	0.461 *p* = 9.4e^−5^	− 0.201 n.s.	0.353 *p* = 0.008	0.69 *p* = 8.1e^−11^	0.508 *p =* 1.5e^−4^	0.423 *p =* 0.02
QUICKI	−0.461 *p* = 9.4e^−5^	0.201 n.s.	−0.353 *p* = 0.008	− 0.69 *p* = 8.1e^−11^	−0.508 *p =* 1.5e^−4^	−0.423 *p =* 0.02
Basal Fructosamine (μM)	0.364 *p =* 0.00*6*	−0.041 n.s	0.152 n.s.	0.177 n.s.	0.124 n.s.	−0.011 n.s.
Basal Grelin (pg/mL)	−0.231 n.s.	−0.373 n.s.	− 0.209 n.s.	−0.464 *p =* 0.034	− 0.348 n.s.	−0.245 n.s.
IGF1 (mg/mL)	0.128 n.s	0.0899 n.s.	0.087 n.s.	0.116 n.s.	0.059 n.s.	0.114 n.s.

*BMI* body mass index, *WC* waist circumference, *HOMA IR* homeostasis model assessment insulin resistance, *QUICKI* quantitative insulin sensitivity check index

Using a stepwise regression method based on AIC (Aikake Information Criterion), we found that the variable that most influenced insulin was BMI (linear regression model: adjusted R^2^ = 0.356; model, p- = 1.591e-09; BMI, *p* = 1.59e-09); the variable that most influenced HOMA-IR was BMI (linear regression model: adjusted R^2^ = 0.3262; model, *p* = 1.036e-08; BMI, *p* = 1.04e-08); and the variables that most influenced BMI were phenylalanine tolerance and age (logistic regression model: null deviance = 110.674 on 82 degrees of fredom, residual deviance = 88.154 on 80 degrees of freedom; Phe tolerance, *p* = 0.00592; age, *p* = 0.00642)

Quick index was significantly lower in patients with CPKU (0.33 ± 0.04) than those with MPKU (0.35 ± 0.04) and MPHA (0.36 ± 0.04) (*p* = 0.026) and was negatively correlated with BMI (rho = − 0.69), WC (rho = − 0.507), age (rho = − 0.46) and annual median blood Phe levels (rho = − 0.35).

When comparing CPKU patients with early and late diagnosis, the late-diagnosis group had higher HOMA-IR values (early diagnosis: 2.97 ± 1.88; late diagnosis: 4.37 ± 2.93) and higher fasting insulin levels (early diagnosis: 14.69 ± 8.7 mIU/L; late diagnosis: 20.36 ± 12.46 mIU/L). The average concentration of HOMA-IR in control group was lower than in PKU patients (1.65 ± 1.29; *p* = 0.0001). In overweight and obese controls (mean age: 25.46 ± 13.77 years, similar age than overweight and obese PKU, *p* = 0.72): insulin and HOMA-IR concentrations also were lower in the controls (insulin: 12.63 ± 5.87 vs 20 ± 9.39 mIU/L; *p* = 0.014; Homa IR 2.86 ± 1.37vs 3.95 ± 2.17, *p* = 0.19).

Using a multiple linear regression model, we assessed the influence of each factor, and found that the variables that most influenced HOMA-IR were BMI and the percentage carbohydrate intake (linear regression model: R^2^ adjusted = 0.352; *p* value of the model = 1.7e^− 5^; p value BMI = 0.0005; p value % carbohydrates = 0.033).

Lactate, C-peptide, IGFBP3, and urea levels were normal in the vast majority of patients. IGF1 levels were elevated in 21 patients (206.6 ± 92.4 mg/mL).Basal ghrelin was high in 28 patients (868.6 ± 453.1 pg/mL) and was negatively correlated with BMI (rho = − 0.46). Fructosamine levels were within normal ranges in 93.98% of patients, but mean levels were significantly higher in adults (239 ± 44 μmol/L vs. 223.6 ± 26 μmol/L, *p =* 0.010).

**Fig. 3:**
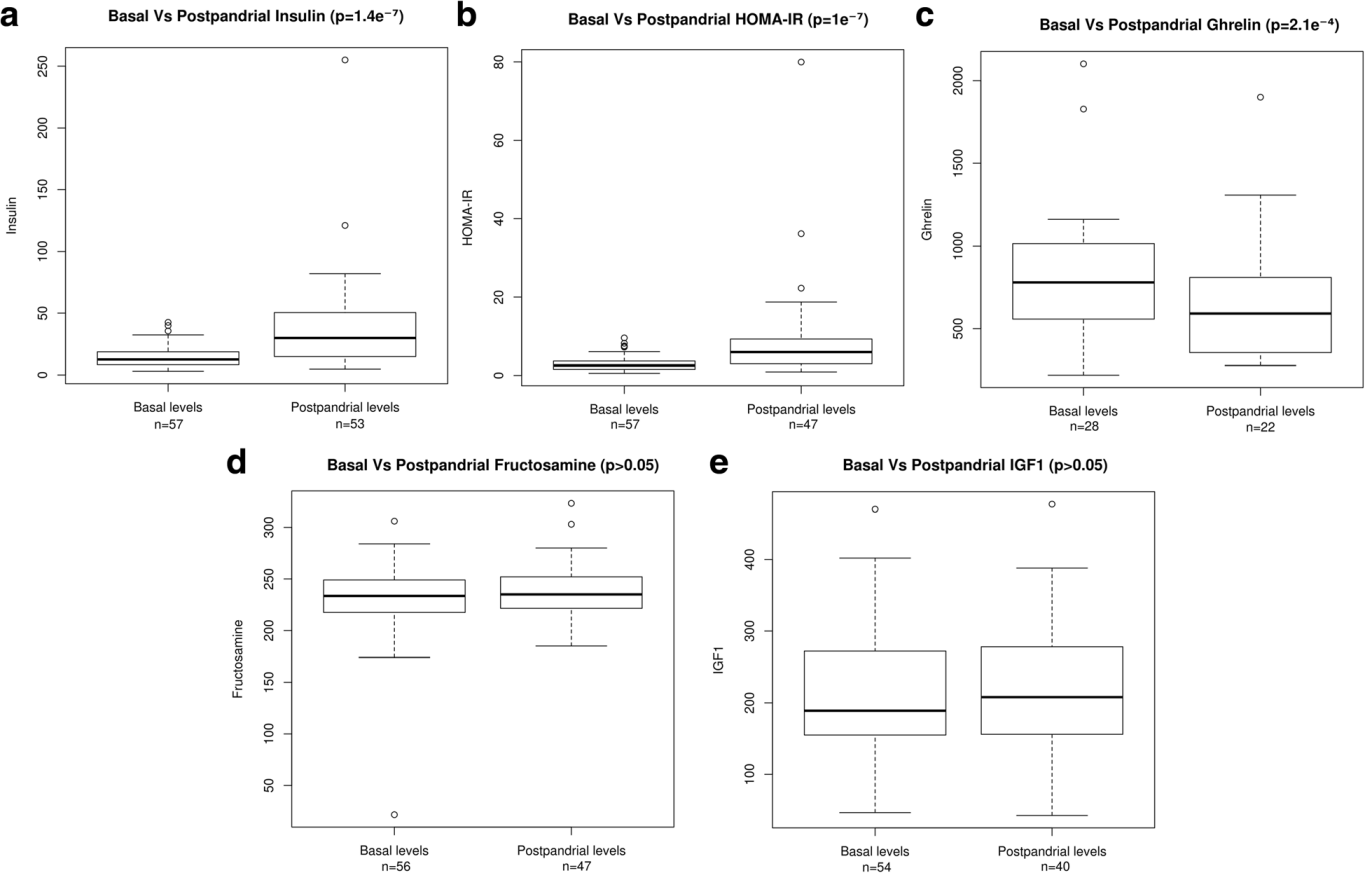
Differences between basal and postpandrial levels in: **a** insulin (mIU/L); **b** HOMA-IR; **c** ghrelin (pg/mL); **d** fructosamine (μmol/L); and **e** IGF1 (mg/mL). HOMA-IR: homeostasis model assessment insulin resistance; IGF1: insulin-like growth factor 1; n: sample size; p: p-value

Caloric intake in the form of carbohydrates was significantly higher in PKU than MHPA patients (*p* = 0.038). It was also positively correlated with basal insulin (rho = 0.47, *p* = 0.006) and HOMA-IR (rho = 0.42, *p* = 0.02) (Table 2). Mean daily carbohydrate intake from AA mixtures was 53.71 ± 9.6 g (range 30–70 g), being higher in CPKU (57.042 ± 8.5 g) than in MPKU patients (53.47 ± 9.8 g). As expected, ingestion of AA mixture led to a marked increase in insulin levels (basal levels: 14.44 ± 9 vs. postprandial levels 37.62 ± 38.4, *p* = 1.4e^− 7^) and HOMA-IR scores (2.9 ± 2 vs. 8.8 ± 12.5, *p* = 1e^− 7^) (Fig. 3). No significant differences were observed for pre- and postprandial fructosamine levels, although ghrelin levels decreased significantly (814.4 ± 416.6 vs. 664.2 ± 379.2; *p* = 0.0002, Fig. 3).

## Discussion

To gain better insights into the metabolic impact of high carbohydrate intake in patients with PKU, we compared carbohydrate metabolism between patients receiving dietary treatment only or dietary and pharmacological treatment for PKU and patients with MPHA on a free diet without excessive intake of proteins of high biological value.

The normal fasting glucose levels found in the majority of patients with PKU were in good agreement with previous findings [14, 17]. However, we observed increased rates of insulin resistance, with higher levels of basal insulin and HOMA-IR in PKU patients, associated with age, BMI, and WC, suggesting possible alterations in glucose homeostasis. This positive correlation between insulin resistance (HOMA-IR) and WC is in line with the findings of Kanufre et al. [16] in children and adolescents with PKU and excess weight. Insulin resistance and basic β-cell dysfunction are primary features of fasting hyperglycemia. A high HOMA index has been associated with an increased prevalence of carbohydrate intolerance and diabetes in obese and non-obese individuals from different ethnic backgrounds [32–35].

Although insulin resistance standards have not been established for the pediatric population [36], mean HOMA-IR in children with PKU in our series (mean ± SD: 2.23 ± 1.51; median (Q1-Q3):1.78(1.47,2.73)was higher than that of pediatric healthy controls (1.57 ± 1.09; in < 7.5 years: 0.61 ± 0.37; in > 7.5 years: 1.84 ± 1.07) and also higher than values reported for the general pediatric population (0.6 [0.3–1.4] for prepubertal children aged < 7.5 years, 1.1 [0.3–2] for those aged > 7.5 years and 1.4 [0.3–2.5] for pubertal adolescents [37]) and for children and adolescents with PKU (median ± SD: 0.9 ± 0.4 [14] and 0.7 ± 1.1 in patients with normal weight [15]). However, comparison with PKU series is difficult since the age of the pediatric patients included varies, ranging from 5 to 11 years [14], 4–15 years [15], and up to 18 years in our study.

The same trend was observed for PKU adults, for whom mean HOMA-IR (3.45 ± 2.19) was higher than that of our adult controls (1.79 ± 1.57) and that reported for the general population in southeast and northeast Spain (1.7 [38] and 1.72 [39], respectively).

HOMA-IR has been independently associated with an increased risk of hypertension [40], and, according to a meta-analysis of 65 studies, this index, together with fasting glucose and insulin levels, is linked to incident cardiovascular disease in individuals without diabetes (46% increase in coronary heart disease risk for an increase of one standard deviation in HOMA-IR) [41].

As previously noted, increased insulin resistance in PKU patients under dietary treatment could be related to metabolic changes caused by long-term intake of high levels of carbohydrate. The deleterious effects of high free sugar-free intake appear to be more evident when this is accompanied by excess energy intake [42]. On average carbohydrates accounted for 35.58% of the total energy content of AA mixtures. This result is in accordance with the data published by Moretti F et al. [14] in a series of PKU children in which Phe-free protein substitutes and special low-protein products accounted for more than 60% of overall daily energy, protein and carbohydrate intake. One limitation of our study is that we were unable to analyze total sugar free intake in PKU patients under dietary treatment as this information is not widely available for the formulas marketed in Spain. According to the Scientific Advisory Committee on Nutrition recommendations on carbohydrates, free sugars should account for no more than 5% of daily dietary energy intake [43].

Insulin resistance is a marker of metabolic syndrome [44, 45] and, while according to the criteria of the American Diabetes Association, it has not yet been linked to pathological glucose levels [46] (with the exception of one case), one could speculate that in the absence of dietary intervention, impaired glucose tolerance could develop at an early age. In our series, insulin resistance was more patent in adults and, coinciding with previous findings [15] in overweight patients.

Although some findings suggest that patients with PKU have similar rates of overweight and obesity to individuals without this disorder [47], a clear tendency towards overweight and obesity (based on BMI) in patients with PKU has also been reported [48–51]. The percentage of overweight/obese subjects in our control group (25%) is similar to that reported for the general population (26.7%) [52]. In our cohort, the percentage of overweight/obese in PKU patients is higher than in HPA (*p* = 0.0062) and healthy controls (*p* = 0.042), especially in those with late diagnosis, probably reflecting poor dietary and exercise habits. However, after adjusting using a logistic regression model, with BMI as the response variable (groups: normal and overweight + obese) and age and diagnosis (groups: PKU and HPA) as explanatory variables, we found that age had the greatest effect on BMI, and that the type of diagnosis alone had no significant effect (age, *p* = 0.0055; type of diagnosis, *p* = 0.0560). Thus, the increased insulin resistance in PKU is likely multifactorial, and particularly influenced by factors such as age, BMI, and dietary carbohydrate intake.

Ghrelin is a growth-hormone-releasing acylated peptide that has been found to enhance appetite [53]. In a study by Weigel et al. [54], 70% of patients with PKU experienced a mean reduction in ghrelin levels of 18.5% (range, 2.2–33%) after ingestion of an AA mixture containing carbohydrate. Although conflicting results have been reported by previous studies of ghrelin in patients with different diseases associated with obesity, our detection of a group of patients with increased basal and postprandial ghrelin levels suggests that homeostatic appetite control mechanisms may be altered in certain cases of PKU, possibly in relation to the nutritional profile of the dietary product prescribed. Although this hypothesis needs to be tested in specifically designed studies, we can speculate that dietary changes might alter the physiological mechanisms of appetite control in certain patients, leading to an increase in central adiposity and insulin resistance.

## Conclusions

Insulin resistance was increased in PKU patients and was particularly evident in adults and in overweight or obese patients. A higher consumption of AA mixture, and a higher caloric intake in the form of carbohydrates, this one positively correlated with fasting insulin and HOMA-IR, was observed in PKU patients, and thus could contribute to carbohydrate intolerance.

## Additional files

Additional file 1: Clinical and basal biochemical characteristics of each patient with hyperphenylalaninemia. (DOCX 48 kb)

Additional file 2: Clinical and biochemical characteristics of healthy controls. (DOCX 21 kb)

## Abbreviations

BMI: Body mass index
CPKU: Classic phenylketonuria
HOMA-IR: Homeostasis Model Assessment Insulin Resistance
IGF1: Insulin-like growth factor 1
IGFBP3: IGF-binding protein 3
LNAA: Large neutral amino acids
MHPA: Mild hyper-Phe
MPKU: Mild-moderate PKU
Phe: Phenylalanine
PKU: Phenylketonuria
WC: Waist circumference
