# Supplementary material for: Carbohydrate status in patients with phenylketonuria
Source: Orphanet J Rare Dis. 2018 Jun 27;13:103. doi: 10.1186/s13023-018-0847-x (PMC6020344; doi:10.1186/s13023-018-0847-x)
Supplement: Supplementary file 1 — Clinical and basal biochemical characteristics of each patient with hyperphenylalaninemia. (DOCX 48 kb) [file 13023_2018_847_MOESM1_ESM.docx]

| P | Age  (y) | Sex | Diag | TD | Tre.  D/P | BMI | WC | Phe  median  µM | Phe tol  mg/dL | Gluc  mg/  dL | Insul  mUI/L | PepC  ng/mL | Urea  mg/  dL | Fruct  µM | Lact  mM | IGF-1  mg/mL | IGF-BP3  µg/mL | Ghrelin  pg/mL |  |  |  |  |  |
| --- | --- | --- | --- | --- | --- | --- | --- | --- | --- | --- | --- | --- | --- | --- | --- | --- | --- | --- | --- | --- | --- | --- | --- |
| 82 | 10y8m | F | CPKU | E | D | N | N | 393 | 490 | 70 | 12.2 | 1.5 | 34 | 195 | 1.4 | 155 | 2.7 | 420 |  |  |  |  |  |
| 19 | 11y1m | M | CPKU | E | D | ↑ | ↑ | 987↑ | 458 | 72 | 8.5 | 1.78 | 18 | 246 | 1.16 | 230 | 5.45 | - |  |  |  |  |  |
| 41 | 11y3m | M | CPKU | E | D | N | N | 200 | 352 | 80 | 7.6 | 1 | 22 | 232 | 1.8 | 173 | 5.2 | 1036↑ |  |  |  |  |  |
| 7 | 12y2m | F | CPKU | E | D | N | N | 290.8 | 337 | 90 | 14.9 | 2.08 | 19 | 248 | 2.38 | 246 | 5.94 | - |  |  |  |  |  |
| 81 | 12y5m | M | CPKU | E | D | ↑ | ↑ | 370 | 390 | 73 | 14.1 | 1.8 | 32 | 210 | 2.1 | 184 | 2.4 | 600 |  |  |  |  |  |
| 14 | 12y9m | M | CPKU | E | D | N | N | 400 | 165 | 92 | 5.8 | 1.44 | 19 | 220 | 0.55 | 273↑ | 4.63 | - |  |  |  |  |  |
| 77 | 13y6m | F | CPKU | E | D | ↑ | ↑ | 229 | 400 | 83 | 20.4↑ | 1.9 | 22 | 237 | 0.54 | 135 | 6.4 | 780↑ |  |  |  |  |  |
| 59 | 14y | M | CPKU | E | D | N | N | 208 | 324 | 99 | 12.1 | 1.5 | 20 | 227 | 0.73 | 340↑ | 5.1 | 697 |  |  |  |  |  |
| 60 | 15y2m | F | CPKU | E | D | ↑ | ↑ | 345 | 327 | 86 | 12.6 | 1.7 | 12 | 246 | 1.09 | 372↑ | 7.1↑ | 344 |  |  |  |  |  |
| 11 | 15y5m | M | CPKU | E | D/P | N | N | 389 | 870 | 76 | 7.5 | 1.7 | 22 | 210 | 0.92 | 168 | 4.2 | - |  |  |  |  |  |
| 80 | 16y2m | F | CPKU | E | D | ↑ | ↑↑ | 410 | 295 | 94 | 32.3↑ | 3.1 | 30 | 235 | 1.8 | 160 | 3.4 | 1100↑ |  |  |  |  |  |
| 31 | 17y1m | F | CPKU | E | D | N | N | 275 | 383 | 72 | 10.6 | 3.2 | 27 | 268 | 0.26 | 346↑ | 5.04 | - |  |  |  |  |  |
| 36 | 17y8m | F | CPKU | E | D | N | N | 413 | 400 | 77 | 8.2 | 1.2 | 19 | 284 | 0.55 | 402↑ | 6.4 | - |  |  |  |  |  |
| 53 | 18y | F | CPKU | E | D | N | N | 557 | 240 | 73 | 7.9 | 1.4 | 20 | 203 | 1.3 | 222 | 5.7 | 666 |  |  |  |  |  |
| 1 | 19y7m | F | CPKU | L | D | ↑↑ | ↑↑ | 230 | 168 | 91 | 42.5↑ | 6.02↑ | 23 | 224 | 1.8 | 170 | 5.93 | - |  |  |  |  |  |
| 8 | 20y7m | M | CPKU | E | D | ↑↑ | ↑↑ | 454 | 488 | 82 | 35.5↑ | 4.38↑ | 31 | 205 | 0.97 | 269↑ | 5.47 | - |  |  |  |  |  |
| 16 | 21y7m | M | CPKU | L | D | N | N | 484 | 490 | 83 | 6.4 | 1.78 | 13 | 260 | 1.33 | 238 | 4.99 | - |  |  |  |  |  |
| 13 | 22y1m | M | CPKU | L | D | ↑ | N | 357 | 420 | 78 | 15.1 | 2.48 | 15 | 250 | 0.73 | 281↑ | 4.34 | - |  |  |  |  |  |
| 68 | 23y11m | M | CPKU | E | D | ↑ | ↑ | 598 | 371 | 80 | 8 | 0.8 | 18 | 274 | 2.07 | 180 | 4.9 | 1014↑ |  |  |  |  |  |
| 18 | 24y2m | M | CPKU | E | D | N | ↑ | 709↑ | 280 | 78 | 18.4 | 3.76 | 18 | 214 | 1.13 | 184 | 4.05 | - |  |  |  |  |  |
| 2 | 24y6m | F | CPKU | E | D | ↑↑ | ↑↑ | 618↑ | 408 | 66 | 28.5↑ | 2.9 | 18 | 222 | 1.14 | 210 | 4.65 | - |  |  |  |  |  |
| 45 | 24y6m | F | CPKU | E | D | ↑ | ↑ | 378 | 237 | 89 | 21.8↑ | 2.3 | 20 |  | 2.53 | 161 | 5.1 | 874↑ |  |  |  |  |  |
| 78 | 24y6m | F | CPKU | E | D | ↑ | ↑ | 378 | 237 | 89 | 21.7↑ | 2.3 | 20 | 240 | 2.53 | 161 | 5.1 | 874↑ |  |  |  |  |  |
| 4 | 26y5m | F | CPKU | E | D | N | ↑ | 1030↑ | 840 | 82 | 9.6 | 1.84 | 23 | 266 | 0.9 | 248 | 5.27 | - |  |  |  |  |  |
| 5 | 26y8m | F | CPKU | E | D | ↑ | ↑↑ | 454 | 210 | 73 | 8.3 | 1.79 | 27 | 266 | 0.79 | 194 | 6.01 | - |  |  |  |  |  |
| 79 | 26y8m | M | CPKU | E | D | ↑ | ↑ | 550 | 318 | 86 | 28.5↑ | 2.4 | 24 | 225 | 1.9 | 175 | 3.1 | 2100↑ |  |  |  |  |  |
| 10 | 31y6m | M | CPKU | E | D | ↑ | ↑↑ | 1254↑ | 273 | 82 | 16.9 | 3.48 | 17 | 278 | 1.32 | 272↑ | 6.7 | - |  |  |  |  |  |
| 17 | 32y7m | F | CPKU | E | D | N | ↑ | 848↑ | 380 | 64 | 3.9 | 1.7 | 22 | 222 | 0.84 | 138 | 3.11 | - |  |  |  |  |  |
| 76 | 32y7m | F | CPKU | E | D | N | ↑ | 840↑ | 380 | 64 | 3.9 | 1.7 | 22 | 22 | 0.84 | 138 | 3.1 | 920↑ |  |  |  |  |  |
| 72 | 34y8m | M | CPKU | E | D | N | ↑ | 848↑ | 330 | 87 | 17 | 1.8 | 26 | 306 | 1.69 | 146 | 4.6 | 825↑ |  |  |  |  |  |
| 74 | 36y11m | M | CPKU | L | D | ↑ | N | 580 | 300 | 88 | 19.6↑ | 3.2 | 24 | 258 | 1.1 | 106 | 4.2 |  |  |  |  |  |  |
| 6 | 41y8m | M | CPKU | L | D | ↑ | ↑ | 969↑ | 350 | 85 | 8.1 | 2.57 | 19 | 233 | 1.1 | 183 | 5.2 | - |  |  |  |  |  |
| 67 | 42y4m | F | CPKU | L | D | ↑↑ | ↑↑ | 787↑ | 612 | 80 | 14 | 2 | 20 | 222 | 1.57 | 140 | 4.3 | 900↑ |  |  |  |  |  |
| 71 | 43y10m | M | CPKU | L | D | ↑ | ↑ | 818↑ | 455 | 75 | 12.4 | 3.3 | 21 | 229 | 1.36 | 86 | 3.3 | 620 |  |  |  |  |  |
| 66 | 43y11m | F | CPKU | L | D | ↑↑ | ↑↑ | 951↑ | 310 | 83 | 39.9↑ | 3.4 | 12 | 234 | 2.09 | en proces | 2.6 | 331 |  |  |  |  |  |
| 70 | 49y9m | M | CPKU | L | D | ↑ | N | 950↑ | 395 | 73 | 18.6↑ | 1.5 | 18 | 250 | 0.7 | 81 | 4 |  |  |  |  |  |  |
| 65 | 52y3m | F | CPKU | L | D | ↑↑ | ↑↑ | 1025↑ | 340 | 111↑ | 27 | 2.5 | 42 | 272 | 0.77 | en proces | 3.6 | 1828↑ |  |  |  |  |  |
| 48 | 4y | M | MPKU | E | D | N | N | 115 | 255 | 74 | 3 | 0.7 | 22 | 188 | 0.84 | 47 | 1.9 | 1180↑ |  |  |  |  |  |
| 47 | 5y | F | MPKU | E | D | N | N | 107 | 233 | 74 | 3.6 | 0.6 | 23 | 174 | 1.24 | 68 | 2.9 | 1016↑ |  |  |  |  |  |
| 37 | 11y1m | F | MPKU | E | D/P | N | N | 152 | 900 | 74 | 3.1 | 0.4 | 24 | 223 | 1.4 | 75 | 3.6 | - |  |  |  |  |  |
| 38 | 11y1m | F | MPKU | E | D/P | N | N | 150 | 950 | 77 | 3.9 | 0.5 | 31 | 194 | 1.6 | 121 | - | - |  |  |  |  |  |
| 47 | 5y | F | MPKU | E | D | N | N | 107 | 233 | 74 | 3.6 | 0.6 | 23 | 174 | 1.24 | 68 | 2.9 | 1016↑ |  |  |  |  |  |
| 52 | 11y8m | M | MPKU | E | D/P | N | N | 223 | 1250 | 78 | 8.3 | 0.9 | 21 | 252 | 1.45 | 170 | 5.2 | 717↑ |  |  |  |  |  |
| 83 | 13y7m | F | MPKU | E | D/P | N | N | 320 | 1500 | 71 | 9.6 | 1.4 | 29 | 180 | 1.5 | 130 | 2.1 | 330 |  |  |  |  |  |
| 55 | 13y8m | F | MPKU | E | D | ↑ | ↑ | 229 | 490 | 83 | 20.4↑ | 1.9 | 22 | 237 | 0.54 | 262↑ | 6.4 | 780↑ |  |  |  |  |  |
| 3 | 15y4m | F | MPKU | E | D | ↑ | N | 448 | 434 | 75 | 15.7 | 3.09 | 18 | 232 | 0.95 | 470↑ | 5.9 | - |  |  |  |  |  |
| 9 | 16y | M | MPKU | E | D | N | N | 218 | 489 | 79 | 8.1 | 1.5 | 13 | 215 | 0.79 | 385 | 6.45 | - |  |  |  |  |  |
| 75 | 16y11m | F | MPKU | E | D | N | N | 504 | 570 | 80 | 10.1 | 1.3 | 21 | 195 | 0.9 | 240 | 5.1 | 220 |  |  |  |  |  |
| 15 | 17y | F | MPKU | E | D | N | N | 521 | 412 | 83 | 12 | 2.58 | 18 | 244 | 0.8 | 275↑ | 5.17 | - |  |  |  |  |  |
| 50 | 17y2m | M | MPKU | E | D/P | N | N | 207.8 | 690 | 72 | 7.9 | 1.2 | 36 | 243 | 1.19 | 260↑ | 6 | 406 |  |  |  |  |  |
| 12 | 17y5m | F | MPKU | L | D | N | N | 250.8 | 339 | 75 | 8.8 | 1.74 | 10 | 234 | 1.7 | 368↑ | 5.77 | - |  |  |  |  |  |
| 34 | 19y2m | F | MPKU | E | D/P | N | N | 281 | 2585 | 82 | 13.1 | 1.5 | 24 | 260 | 1.79 | 295↑ | 6 | - |  |  |  |  |  |
| 57 | 19y3m | M | MPKU | E | D/P | N | N | 233 | 1500 | 81 | 10.6 | 1.4 | 24 | 245 | 0.59 | 194 | 4 | 516 |  |  |  |  |  |
| 32 | 20y5m | M | MPKU | E | D | N | N | 684↑ | 496 | 81 | 14.8 | 1.6 | 33 | 239 | 0.24 | 275↑ | 3.08 | - |  |  |  |  |  |
| 58 | 23y1m | F | MPKU | L | D/P | ↑ | ↑ | 199 | 2500 | 77 | 13.3 | 1.7 | 27 | 244 | 0.52 | 218 | 4.7 | 1037↑ |  |  |  |  |  |
| 33 | 27y8m | F | MPKU | E | D | ↑ | N | 969↑ | 495 | 86 | 13.5 | 1.6 | 29 | 220 | 0.21 | 297↑ | 5.01 | - |  |  |  |  |  |
| 20 | 29y9m | F | MPKU | E | D | ↑↑ | ↑↑ | 610↑ | 490 | 77 | 20.4↑ | 3.35 | 22 | 208 | 1.14 | 164 | 4.38 | - |  |  |  |  |  |
| 69 | 31y5m | M | MPKU | E | D/P | ↑ | ↑ | 475 | 800 | 82 | 22.3↑ | 1.9 | 19 | 229 | 1.71 | 137 | 4.6 | 693 |  |  |  |  |  |
| 30 | 2y4m | M | MHPA | E | - | N | N | 190 | free | 83 | 7.8 | 1.4 | 19 | 212 | 3.8 | 163 | 2.04 | - |  |  |  |  |  |
| 54 | 4y | F | MHPA | E | - | ↓ | ↓ | 320 | free | 81 | 5 | 0.7 | 23 | 194 | 1.16 | 61 | 3 | 1190↑ |  |  |  |  |  |
| 51 | 3y 9m | M | MHPA | E | - | ↑ | ↑ | 310 | free | 64 | 1.8 | 0.3 | 20 | 227 | 2.45 | 41 | 2.4 | 1062↑ |  |  |  |  |  |
| 46 | 5y 5m | F | MHPA | E | - | N | N | 222 | free | 76 | 2.4 | 0.5 | 23 | 232 | 1.47 | 94 | 5.1 | 1160↑ |  |  |  |  |  |
| 39 | 6y 2m | F | MHPA | E | - | N | N | 151 | free | 81 | 4.4 | 0.6 | 39 | 215 | 1.37 | 150 | 5.2 | 936↑ |  |  |  |  |  |
| 64 | 6y 8m | M | MHPA | E | - | N | N | 190 | free | 87 | 12.1 | 1 | 23 | 252 | - | 121 | 4 | 2334↑ |  |  |  |  |  |
| 62 | 7y 3m | M | MHPA | E | - | ↑ | ↑ | 270 | free | 82 | 8.8 | 1.3 | 34 | 204 | 1.34 | 152 | 3.1 | 834↑ |  |  |  |  |  |
| 56 | 7y 4m | F | MHPA | E | - | N | N | 289 | free | 73 | 4.5 | 0.4 | 31 | 230 | 1.03 | 74 | 3.7 | 1185↑ |  |  |  |  |  |
| 27 | 7y 8m | M | MHPA | E | - | N | N | 185 | free | 84 | 5.3 | 1.12 | 40 | 212 | 0.77 | 162 | 4.24 | - |  |  |  |  |  |
| 40 | 7y 11m | F | MHPA | E | - | N | ↓ | 193 | free | 78 | 6.4 | 0.7 | 24 | 303 | 1.2 | 150 | 4.5 | 961↑ |  |  |  |  |  |
| 42 | 8y 7m | F | MHPA | E | - | N | N | 310 | free | 66 | 6.3 | 0.7 | 28 | 197 | 1.22 | 117 | 3.5 | 667 |  |  |  |  |  |
| 35 | 9y 3m | M | MHPA | E | - | N | N | 305 | free | 88 | 5.6 | 0.8 | 30 | 237 | 1.21 | 211 | 4.3 | - |  |  |  |  |  |
| 23 | 11y | F | MHPA | E | - | N | N | 315 | free | 83 | 14.2 | 2.21 | 14 | 235 | 1.14 | 410↑ | 5.17 | - |  |  |  |  |  |
| 63 | 11y 2m | F | MHPA | E | - | N | N | 240 | free | 92 | 21.2↑ | 2.9 | 29 | 188 | 1.86 | 652↑ | 5.2 | 601 |  |  |  |  |  |
| 49 | 11y 5m | F | MHPA | E | - | N | N | 295 | free | 80 | 10.7 | 1 | 20 | 188 | 0.9 | 182 | 3.6 |  |  |  |  |  |  |
| 44 | 11y 6m | F | MHPA | E | - | N | N | 299 | free | 80 | 8.2 | 0.6 | 24 | 242 | 0.84 | 173 | 4.7 | 1060↑ |  |  |  |  |  |
| 21 | 12y 9m | M | MHPA | E | - | N | N | 351 | free | 87 | 6 | 1.07 | 25 | 232 | 1.46 | 156 | 4.72 | - |  |  |  |  |  |
| 43 | 13y 1m | M | MHPA | E | - | ↑↑ | ↑ | 297 | free | 88 | 19.3↑ | 1.6 | 35 | 196 | 1.19 | 167 | 4.5 | 109 |  |  |  |  |  |
| 22 | 13y 4m | F | MHPA | E | - | N | N | 266 | free | 83 | 8 | 1.83 | 19 | 246 | 0.92 | 347↑ | 5.29 | - |  |  |  |  |  |
| 28 | 20y11m | F | MHPA | E | - | N | N | 339 | free | 91 | 11.9 | 1.91 | 17 | 258 | 1.08 | 236 | 4.82 | - |  |  |  |  |  |
| 73 | 21y11m | F | MHPA | E | - | ↑ | ↑ | 320 | free | 86 | 21.5↑ | 2 | 29 | 224 | 0.93 | 142 | 5.3 | 74 |  |  |  |  |  |
| 25 | 22y 6m | F | MHPA | E | - | N | N | 290 | free | 99 | 7.9 | 2.52 | 27 | 210 | 1.2 | 232 | 4.38 | - |  |  |  |  |  |
| 26 | 24y 6m | M | MHPA | E | - | N | N | 297 | free | 84 | 9 | 1.6 | 33 | 289 | 0.82 | 298↑ | 5.53 | - |  |  |  |  |  |
| 24 | 24y 7m | F | MHPA | E | - | N | N | 296 | free | 77 | 14 | 2.52 | 25 | 257 | 0.9 | 248 | 5.73 | - |  |  |  |  |  |
| 29 | 31y 4m | F | MHPA | E | - | N | N | 365 | free | 83 | 8.2 | 1.8 | 21 | 266 | 0.95 | 183 | 5.33 | - |  |  |  |  |  |
| 61 | 47y 3m | F | MHPA | L | - | N | N | 290 | free | 81 | 4.3 | 0.9 | 28 | 303 | 0.93 | 105 | 4.7 | 1150↑ |  |  |  |  |  |

Additional file 1. Clinical and basal biochemical characteristics of each patient with hyperphenylalaninemia

P: patient; Y: years; M: male; F: female; CPKU: classic PKU; MPKU: mild-moderate PKU; MHPA: mild hyperphenylalaninemia; TD: time of diagnosis; L: late; E: early; Tre: Treatment; D: dietary treatment; P: pharmacological treatment; BMI: body mass index; WC: waist circumference; N: normal; ↑: overweight; ↑↑: Obesity; Median Phe: annual median blood Phe levels; Phe tol: Phenylalanine tolerance; Gluc: glucose; Insul: insulin; Fruct: fructosamine; Lact: lactate.
