# Supplementary material for: Carbohydrate status in patients with phenylketonuria
Source: Orphanet J Rare Dis. 2018 Jun 27;13:103. doi: 10.1186/s13023-018-0847-x (PMC6020344; doi:10.1186/s13023-018-0847-x)
Supplement: Supplementary file 2 — Clinical and biochemical characteristics of healthy controls. (DOCX 21 kb) [file 13023_2018_847_MOESM2_ESM.docx]

| Controls  (n) | Age (y) | Sex | BMI | WC | Glucose  mg/dL | Insulin  mUI/L |
| --- | --- | --- | --- | --- | --- | --- |
| 1 | 32y | F | N | N | 93 | 7.4 |
| 2 | 37y | M | N | N | 96 | 3.3 |
| 3 | 27y | F | N | N | 80 | 2 |
| 4 | 24y | F | N | N | 94 | 16.5 |
| 5 | 33y | M | ↑ | N | 71↓ | 2 |
| 6 | 27y | F | N | N | 73↓ | 2 |
| 7 | 25y | F | N | N | 73↓ | 2.2 |
| 8 | 33y | F | N | N | 78 | 2 |
| 9 | 31y | F | N | N | 75 | 2 |
| 10 | 28y | F | N | N | 92 | 2 |
| 11 | 38y | M | N | N | 87.29 | 4.45 |
| 12 | 34y | M | N | N | 102.11 | 6.02 |
| 13 | 44y | M | N | N | 97.97 | 3.08 |
| 14 | 42y | F | N | N | 91.25 | 3.65 |
| 15 | 49y | F | N | N | 95.48 | 4.09 |
| 16 | 36y | F | ↑ | N | 91.23 | 5.46 |
| 17 | 34y8m | M | ↑↑ | ↑↑ | 89 | 20.9↑ |
| 18 | 46y7m | F | ↑↑ | ↑↑ | 85 | 14.9 |
| 19 | 49y2m | F | ↑↑ | ↑↑ | 111↑ | 12.4 |
| 20 | 43y10m | M | ↑↑ | ↑↑ | 99 | 23.9↑ |
| 21 | 31y3m | M | ↑↑ | ↑ | 102 | 10.2 |
| 22 | 33y6m | F | ↑↑ | ↑↑ | 91 | 17.5 |
| 23 | 18y3m | F | ↑↑ | ↑↑ | 79 | 17.1 |
| 24 | 10y8m | F | N | N | 91 | 4.60 |
| 25 | 11y1m | M | N | N | 81 | 2.79 |
| 26 | 11y4m | M | N | N | 82 | 5.70 |
| 27 | 12y2m | F | N | N | 78 | 20.30↑ |
| 28 | 12y3m | M | ↑↑ | ↑↑ | 85 | 12.00 |
| 29 | 12y8m | M | N | N | 89 | 1.99 |
| 30 | 13y6m | F | ↑ | ↑ | 93 | 13.80 |
| 31 | 14y2m | M | N | N | 92 | 9.90 |
| 32 | 15y | F | N | N | 76 | 2.70 |
| 33 | 15y6m | M | N | N | 93 | 7.40 |
| 34 | 16y2m | M | ↑ | ↑ | 91 | 5.00 |
| 35 | 16y10m | M | N | ↑ | 93 | 2.60 |
| 36 | 17y1m | M | N | N | 72↓ | - |
| 37 | 18y6m | M | N | N | 82 | 6.90 |
| 38 | 4y1m | F | N | N | 79 | 1.90 |
| 39 | 5y1m | F | ↑ | N | 79 | 7.70 |
| 40 | 11y1m | F | N | - | 84 | 9.30 |
| 41 | 11y1m | F | N | N | 89 | 8.60 |
| 42 | 5y1m | M | N | N | 82 | 2.00 |
| 43 | 11y9m | F | N | N | 82 | 5.20 |
| 44 | 13y6m | M | ↑↑ | ↑↑ | 86 | 17.80 |
| 45 | 14y1m | F | N | ↑ | 78 | 9.30 |
| 46 | 15y9m | F | ↑↑ | ↑ | 91 | 7.80 |
| 47 | 16y1m | M | N | ↑ | 93 | 14.00 |
| 48 | 16y9m | F | ↑ | ↑ | 90 | 14.20 |
| 49 | 17y1m | M | N | N | 72↓ | - |
| 50 | 18y6m | M | N | N | 82 | 6.90 |
| 51 | 4y1m | M | N | N | 82 | 2.00 |
| 52 | 4y8m | M | N | N | 71↓ | 1.99 |
| 53 | 5y4m | M | N | N | 77 | 4.60 |
| 54 | 6y2m | F | N | N | 87 | - |
| 55 | 6y9m | M | N | N | 78 | 3.10 |
| 56 | 7y3m | F | N | N | 79 | 2.30 |
| 57 | 7y3m | M | N | N | 89 | 2.50 |
| 58 | 7y9m | M | N | N | 77 | 4.03 |
| 59 | 7y10m | F | N | N | 86 | 9.10 |
| 60 | 8y8m | F | N | N | 77 | 3.44 |
| 61 | 9y3m | F | N | N | 77 | 4.60 |
| 62 | 11y | F | N | N | 88 | 13.80 |
| 63 | 11y2m | M | N | N | 87 | 5.60 |
| 64 | 11y6m | F | N | N | 87 | 4.20 |
| 65 | 11y7m | F | N | N | 91 | 10.60 |
| 66 | 12y10m | F | N | N | 75 | 7.00 |
| 67 | 13y1m | M | ↑ | ↑ | 108↑ | 12.10 |
| 68 | 13y3m | F | N | ↑ | 85 | 16.30 |

Additional file 2. Clinical and biochemical characteristics of healthy controls.

N. number; Y: years; M: male, F: female; BMI: body mass index; WC: waist circumference; N: normal; ↑: overweight; ↑↑: Obesity.
